# COVID-19 Vaccination Coverage, by Race and Ethnicity — National Immunization Survey Adult COVID Module, United States, December 2020–November 2021

**DOI:** 10.15585/mmwr.mm7123a2

**Published:** 2022-06-10

**Authors:** Jennifer L. Kriss, Mei-Chuan Hung, Anup Srivastav, Carla L. Black, Megan C. Lindley, James T. Lee, Ram Koppaka, Yuping Tsai, Peng-Jun Lu, David Yankey, Laurie D. Elam-Evans, James A. Singleton

**Affiliations:** ^1^CDC COVID-19 Emergency Response Team; ^2^Immunization Services Division, National Center for Immunization and Respiratory Diseases, CDC; ^3^Leidos, Inc., Reston, Virginia.

Some racial and ethnic minority groups have experienced disproportionately higher rates of COVID-19–related illness and mortality (*1*,*2*). Vaccination is highly effective in preventing severe COVID-19 illness and death (*3*), and equitable vaccination can reduce COVID-19–related disparities. CDC analyzed data from the National Immunization Survey Adult COVID Module (NIS-ACM), a random-digit–dialed cellular telephone survey of adults aged ≥18 years, to assess disparities in COVID-19 vaccination coverage by race and ethnicity among U.S. adults during December 2020–November 2021. Asian and non-Hispanic White (White) adults had the highest ≥1-dose COVID-19 vaccination coverage by the end of April 2021 (69.6% and 59.0%, respectively); ≥1-dose coverage was lower among Hispanic (47.3%), non-Hispanic Black or African American (Black) (46.3%), Native Hawaiian or other Pacific Islander (NH/OPI) (45.9%), multiple or other race (42.6%), and American Indian or Alaska Native (AI/AN) (38.7%) adults. By the end of November 2021, national ≥1-dose COVID-19 vaccination coverage was similar for Black (78.2%), Hispanic (81.3%), NH/OPI (75.7%), and White adults (78.7%); however, coverage remained lower for AI/AN (61.8%) and multiple or other race (68.0%) adults. Booster doses of COVID-19 vaccine are now recommended for all adults (*4*), but disparities in booster dose coverage among the fully vaccinated have become apparent (*5*). Tailored efforts including community partnerships and trusted sources of information could be used to increase vaccination coverage among the groups with identified persistent disparities and can help achieve vaccination equity and prevent new disparities by race and ethnicity in booster dose coverage.

NIS-ACM is a random-digit–dialed cellular telephone survey of adults aged ≥18 years in all 50 states, the District of Columbia, and selected local areas and U.S. territories.* Data are weighted to represent the noninstitutionalized U.S. adult population and calibrated to state-level vaccine administration data reported to CDC.^†^ Survey respondents who reported their race and ethnicity^§^ and whether they received ≥1 dose of COVID-19 vaccine^¶^ (516,190) during April 22–December 31, 2021, were included; race and ethnicity was reported for 97.1% of respondents. First-dose vaccination month and year were imputed for 4.9% of persons who reported they received vaccination but did not report their month and year of vaccination, using hot deck imputation (replacing missing values with observed values from a respondent with similar characteristics) from donor pools matched for month of interview, age group, region, and race and ethnicity. Monthly survey response rates ranged from 17.2% to 23.4% (average = 20.6%).**

The Kaplan-Meier survival analysis procedure was used, with vaccination month as the time-to-event variable, to estimate the cumulative percentage of persons vaccinated by the end of each month during December 2020–November 2021.^††^ Differences in ≥1-dose COVID-19 vaccination coverage were assessed by race and ethnicity and stratified by U.S. Census Bureau region,^§§^ urbanicity,^¶¶^ age group, annual household income, and health insurance status. T-tests were used to determine differences among groups, with p<0.05 considered statistically significant. Analyses were conducted using SAS (version 9.4; SAS Institute) and SUDAAN (version 11; RTI International). This activity was reviewed by CDC and was conducted consistent with applicable federal law and CDC policy.***

By the end of April 2021, when all U.S. adults were eligible to receive COVID-19 vaccine, vaccination coverage was highest among adults who were Asian (69.6%) or White (59.0%), and lower among those who were Hispanic (47.3%), Black (46.3%), NH/OPI (45.9%), multiple or other race (42.6%), or AI/AN (38.7%) (Figure). Differences in coverage among these racial and ethnic groups compared with that in White adults peaked during March–May 2021, after which disparities began to diminish (Figure). By the end of November 2021, differences in vaccination coverage were no longer statistically significant among Black and NH/OPI adults (difference = −0.5 and −3.0 percentage points, respectively), compared with coverage among White adults (Table 1). Vaccination coverage among Hispanic and Asian adults was higher than coverage among White adults (difference = 2.6 and 16.5 percentage points, respectively), whereas coverage remained lower among AI/AN (difference = −16.9) and multiple or other race (difference = −10.7) adults.

**FIGURE:**
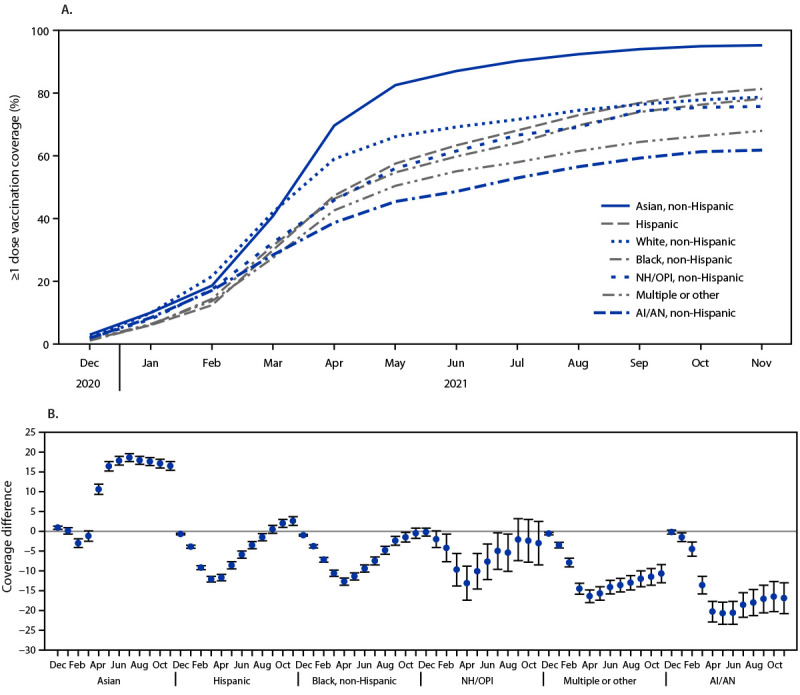
COVID-19 vaccination (≥1 dose) coverage estimates (A)* among adults aged ≥18 years, by race and ethnicity and differences in coverage from White, non-Hispanic adults, and by race and ethnicity (B)^†^^,^^§^^,^^¶^ — National Immunization Survey Adult COVID Module, United States, December 2020–November 2021 **Abbreviations:** AI/AN = American Indian or Alaska Native; NH/OPI = Native Hawaiian or other Pacific Islander. * Kaplan-Meier survival analysis was used to estimate vaccination coverage based on the month and year of first dose receipt; estimates reflect the cumulative percentage vaccinated as of the end of each month. ^†^ Referent group = White, non-Hispanic. ^§^ Persons were categorized into mutually exclusive categories of race and ethnicity; persons who did not identify as Hispanic were categorized by their reported race or races. ^¶^ 95% CIs indicated by error bars.

**TABLE 1:** Differences in COVID-19 vaccination (≥1 dose) coverage,* by race and ethnicity^†^ and selected geographic and sociodemographic characteristics — National Immunization Survey Adult COVID Module, United States, April 2021 and November 2021

Characteristic	Coverage (95% CI)	Coverage difference (95% CI)					
	White, non-Hispanic (ref) n = 329,135	Black, non-Hispanic n = 61,848	Hispanic n = 67,925	AI/AN n = 6,224	Asian n = 26,468	NH/OPI n = 5,149	Multiple or other race n = 19,441
**April 2021**							
**Overall**	**59.0 (58.6 to 59.3)**	**−12.7 (−13.6 to −11.8)^§^**	**−11.7 (−12.5 to −10.9)^§^**	**−20.3 (−22.9 to −17.7)^§^**	**10.6 (9.3 to 11.9)^¶^**	**−13.1 (−17.4 to −8.8)^§^**	**−16.4 (−18.0 to −14.8)^§^**
**U.S. Census Bureau region**							
Northeast	69.0 (68.3 to 69.7)	−21.2 (−23.1 to −19.3)^§^	−22.8 (−24.6 to −21.0)^§^	−31.3 (−38.6 to −24.1)^§^	−1.4 (−3.6 to 0.8)	−16.8 (−28.1 to −5.6)^§^	−18.3 (−21.6 to −15.0)^§^
Midwest	55.9 (55.1 to 56.7)	−13.1 (−15.4 to −10.8)^§^	−14.5 (−16.7 to −12.3)^§^	−22.6 (−28.5 to −16.7)^§^	10.0 (6.7 to 13.4)**^¶^**	−30.5 (−39.4 to −21.6)^§^	−20.2 (−23.8 to −16.7)^§^
South	53.7 (53.1 to 54.3)	−7.2 (−8.4 to −6.1)^§^	−8.6 (−10.0 to −7.2)^§^	−21.4 (−25.0 to −17.8)^§^	12.8 (10.3 to 15.2)**^¶^**	−18.0 (−27.5 to −8.5)^§^	−18.9 (−21.1 to −16.6)^§^
West	63.0 (62.2 to 63.8)	−13.3 (−16.4 to −10.2)^§^	−13.9 (−15.5 to −12.3)^§^	−16.3 (−21.1 to −11.6)^§^	9.6 (7.5 to 11.8)**^¶^**	−13.3 (−19.2 to −7.5)^§^	−12.8 (−16.0 to −9.7)^§^
Urbanicity**							
MSA, principal city	63.4 (62.7 to 64.1)	−19.9 (−21.3 to −18.5)^§^	−15.9 (−17.2 to −14.5)^§^	−27.8 (−32.7 to −22.9)^§^	5.3 (3.4 to 7.2)**^¶^**	−14.2 (−21.5 to −7.0)^§^	−19.3 (−22.0 to −16.6)^§^
MSA, nonprincipal city	60.1 (59.6 to 60.5)	−11.4 (−12.6 to −10.1)^§^	−12.3 (−13.4 to −11.1)^§^	−22.3 (−26.4 to −18.2)^§^	11.1 (9.3 to 12.9)**^¶^**	−16.3 (−22.4 to −10.1)^§^	−16.3 (−18.5 to −14.0)^§^
Non-MSA	49.1 (48.2 to 49.9)	−2.7 (−5.4 to 0.1)	−7.1 (−9.7 to −4.4)^§^	−5.6 (−10.0 to −1.2)^§^	6.3 (−1.1 to 13.7)	−4.0 (−14.6 to 6.5)	−14.5 (−18.1 to −10.8)^§^
**Age group, yrs**							
18–29	35.9 (35.1 to 36.7)	−13.8 (−15.4 to −12.2)^§^	−2.6 (−4.1 to −1.2)^§^	−13.8 (−18.6 to −9.0)^§^	24.1 (21.7 to 26.5)**^¶^**	−14.9 (−20.8 to −8.9)^§^	−3.0 (−5.7 to −0.3)^§^
30–49	48.5 (47.9 to 49.1)	−12.7 (−14.0 to −11.3)^§^	−3.8 (−5.1 to −2.5)^§^	−13.9 (−18.0 to −9.9)^§^	21.9 (20.0 to 23.8)**^¶^**	−3.3 (−9.7 to 3.0)	−11.5 (−14.0 to −9.0)^§^
50–64	63.1 (62.5 to 63.8)	−6.6 (−8.2 to −4.9)^§^	−3.3 (−5.1 to −1.5)^§^	−20.0 (−24.9 to −15.1)^§^	14.5 (11.6 to 17.4)**^¶^**	−9.6 (−18.7 to −0.4)^§^	−16.6 (−20.0 to −13.2)^§^
≥65	82.8 (82.3 to 83.4)	−8.5 (−10.3 to −6.7)^§^	−7.4 (−9.5 to −5.4)^§^	−21.9 (−29.9 to −13.9)^§^	2.7 (−0.5 to 5.9)	0.2 (−8.0 to 8.4)	−12.9 (−16.4 to −9.4)^§^
**Annual household income**							
Below poverty	36.5 (35.3 to 37.7)	−2.5 (−4.8 to −0.2)^§^	3.4 (1.3 to 5.4)**^¶^**	−3.5 (−9.5 to 2.4)	21.6 (17.2 to 26.0)**^¶^**	−9.2 (−18.3 to 0.1)	−11.6 (−15.2 to −8.1)^§^
Above poverty, <$75,000	54.6 (54.0 to 55.3)	−10.1 (−11.6 to −8.7)^§^	−8.1 (−9.5 to −6.7)^§^	−16.5 (−20.5 to −12.4)^§^	10.9 (8.3 to 13.6)**^¶^**	−7.8 (−15.4 to −0.2)^§^	−14.4 (−17.0 to −11.8)^§^
Above poverty, ≥$75,000	67.6 (67.1 to 68.2)	−9.5 (−11.3 to −7.8)^§^	−9.8 (−11.5 to −8.2)^§^	−19.5 (−24.9 to −14.1)^§^	9.1 (7.3 to 10.8)**^¶^**	−14.6 (−22.4 to −6.8)^§^	−12.6 (−15.6 to −9.7)^§^
Unknown	58.6 (57.8 to 59.4)	−13.1 (−14.9 to −11.4)^§^	−14.8 (−16.5 to −13.1)^§^	−21.7 (−27.3 to −16.1)^§^	6.9 (4.2 to 9.7)**^¶^**	−13.1 (−22.5 to −3.7)^§^	−16.5 (−19.9 to −13.1)^§^
**Health insurance**							
Insured	61.6 (61.2 to 61.9)	−12.2 (−13.1 to −11.2)^§^	−9.6 (−10.5 to −8.6)^§^	−20.6 (−23.4 to −17.7)^§^	10.0 (8.7 to 11.3)**^¶^**	−11.3 (−16.0 to −6.6)^§^	−15.7 (−17.4 to −14.0)^§^
Not insured	28.0 (26.9 to 29.2)	−4.3 (−6.5 to −2.0)^§^	3.0 (1.1 to 4.9)**^¶^**	−6.4 (−12.3 to −0.5)^§^	23.5 (18.6 to 28.3)**^¶^**	−7.4 (−15.4 to 0.5)	−8.2 (−11.9 to −4.5)^§^
**November 2021**							
**Overall**	78.7 (78.2 to 79.1)	−0.5 (−1.8 to 0.8)	2.6 (1.5 to 3.7)**^¶^**	−16.9 (−20.8 to −13.0)^§^	16.5 (15.4 to 17.6)**^¶^**	−3.0 (−8.5 to 2.5)	−10.7 (−13.0 to −8.4)^§^
**U.S. Census Bureau region**							
Northeast	88.0 (87.2 to 88.8)	−2.4 (−4.9 to 0.1)	−2.0 (−4.2 to 0.2)	−12.6 (−24.3 to −0.9)^§^	8.2 (6.6 to 9.8)**^¶^**	1.7 (−6.9 to 10.3)	−5.9 (−10.6 to −1.3)^§^
Midwest	74.0 (73.0 to 74.9)	−5.4 (−8.6 to −2.2)^§^	−3.7 (−6.6 to −0.7)^§^	−24.4 (−33.0 to −15.8)^§^	16.9 (12.8 to 21.1)**^¶^**	−24.3 (−43.3 to −5.3)^§^	−19.5 (−24.2 to −14.9)^§^
South	74.1 (73.4 to 74.9)	4.6 (2.9 to 6.4)**^¶^**	4.4 (2.4 to 6.4)**^¶^**	−20.8 (−26.5 to −15.0)^§^	20.1 (17.9 to 22.3)**^¶^**	−19.0 (−31.5 to −6.5)^§^	−13.9 (−18.2 to −9.5)^§^
West	84.3 (83.4 to 85.2)	−3.8 (−8.1 to 0.6)	−1.3 (−3.3 to 0.7)	−12.1 (−19.0 to −5.2)^§^	11.8 (10.0 to 13.6)**^¶^**	−3.0 (−9.7 to 3.7)	−8.4 (−12.4 to −4.4)^§^
Urbanicity**							
MSA, principal city	83.2 (82.4 to 84.0)	−6.6 (−8.5 to −4.6)^§^	−0.8 (−2.6 to 1.0)	−21.6 (−28.7 to −14.4)^§^	12.5 (10.9 to 14.1)**^¶^**	−4.0 (−11.1 to 3.1)	−10.9 (−14.6 to −7.2)^§^
MSA, nonprincipal city	79.5 (78.9 to 80.1)	−0.2 (−1.9 to 1.6)	2.1 (0.6 to 3.6)**^¶^**	−23.1 (−28.9 to −17.3)^§^	15.7 (14.1 to 17.3)**^¶^**	−5.1 (−13.3 to 3.2)	−9.5 (−13.1 to −5.9)^§^
Non-MSA	69.4 (68.2 to 70.5)	10.3 (5.1 to 15.5)**^¶^**	2.3 (−1.5 to 6.2)	0.1 (−6.6 to 6.9)	19.2 (10.7 to 27.7)**^¶^**	0.4 (−15.0 to 15.8)	−18.7 (−23.4 to −14.0)^§^
**Age group, yrs**							
18–29	62.5 (61.3 to 63.8)	−4.2 (−7.6 to −0.7)^§^	9.0 (6.6 to 11.5)**^¶^**	−17.3 (−25.5 to −9.1)^§^	30.2 (27.7 to 32.8)**^¶^**	−14.1 (−25.4 to −2.8)^§^	−0.4 (−5.1 to 4.4)
30–49	71.2 (70.3 to 72.1)	1.1 (−1.4 to 3.6)	9.0 (7.1 to 10.8)**^¶^**	−15.7 (−21.5 to −10.0)^§^	24.3 (22.5 to 26.1)**^¶^**	3.0 (−4.8 to 10.7)	−8.9 (−12.7 to −5.2)^§^
50–64	83.0 (82.2 to 83.8)	5.6 (3.6 to 7.6)**^¶^**	7.6 (5.8 to 9.4)**^¶^**	−14.7 (−22.4 to −7.1)^§^	15.1 (13.9 to 16.3)**^¶^**	10.4 (5.4 to 15.4)**^¶^**	−12.1 (−16.5 to −7.8)^§^
≥65	94.1 (93.6 to 94.6)	−1.2 (−2.8 to 0.4)	1.2 (−0.2 to 2.5)	−8.4 (−18.9 to 2.2)	3.5 (1.6 to 5.4)**^¶^**	5.5 (4.6 to 6.4)**^¶^**	−2.9 (−8.2 to 2.5)
**Annual household income**							
Below poverty	64.8 (62.8 to 66.8)	4.8 (1.1 to 8.5)**^¶^**	15.2 (12.1 to 18.3)**^¶^**	−2.6 (−12.0 to 6.8)	29.8 (26.3 to 33.3)**^¶^**	−2.5 (−18.5 to 13.6)	−8.9 (−15.7 to −2.2)^§^
Above poverty, <$75,000	76.4 (75.6 to 77.2)	1.3 (−0.9 to 3.5)	4.6 (2.7 to 6.5)**^¶^**	−14.3 (−20.6 to −7.9)^§^	17.9 (15.7 to 20.0)**^¶^**	2.5 (−7.4 to 12.4)	−10.7 (−14.7 to −6.8)^§^
Above poverty, ≥$75,000	84.3 (83.7 to 84.9)	0.6 (−1.5 to 2.7)	−0.1 (−2.0 to 1.8)	−14.2 (−20.7 to −7.8)^§^	13.3 (12.2 to 14.5)**^¶^**	−2.5 (−10.1 to 5.1)	−8.3 (−12.0 to −4.7)^§^
Unknown	77.4 (76.4 to 78.3)	0.5 (−2.2 to 3.2)	2.2 (−0.2 to 4.7)	−21.4 (−29.5 to −13.2)^§^	15.1 (12.2 to 18.0)**^¶^**	−7.1 (−18.0 to 3.8)	−8.2 (−13.6 to −2.8)^§^
**Health insurance**							
Insured	80.7 (80.3 to 81.2)	0.0 (−1.4 to 1.3)	2.9 (1.8 to 4.1)**^¶^**	−16.2 (−20.5 to −12.0)^§^	15.2 (14.1 to 16.3)**^¶^**	−3.7 (−9.0 to 1.6)	−8.8 (−11.3 to −6.4)^§^
Not insured	53.9 (51.9 to 55.9)	4.7 (0.3 to 9.1)**^¶^**	19.3 (15.9 to 22.6)**^¶^**	−11.5 (−21.2 to −1.7)^§^	33.3 (27.4 to 39.2)**^¶^**	19.5 (0.5 to 38.5)**^¶^**	−13.8 (−20.1 to −7.6)^§^

**Abbreviations:** AI/AN = American Indian or Alaska Native; MSA = metropolitan statistical area; NH/OPI = Native Hawaiian or other Pacific Islander; Ref = referent group.

* Kaplan-Meier survival analysis was used to estimate vaccination coverage based on the month and year of first dose receipt; estimates reflect the cumulative percentage vaccinated as of the end of each month.

^†^ Persons were categorized into mutually exclusive categories of race and ethnicity; persons who did not identify as Hispanic were categorized by their reported race or races.

^§^ Coverage is statistically significantly lower than coverage among White, non-Hispanic adults (p<0.05).

^¶^ Coverage is statistically significantly higher than coverage among White, non-Hispanic adults (p<0.05).

** MSA status was determined based on household reported city and county of residence and was grouped into three categories: MSA principal city (urban), MSA nonprincipal city (suburban), and non-MSA (rural). MSAs and principal cities were as defined by the U.S. Census Bureau (https://www.census.gov/programs-surveys/metro-micro.html). Non-MSA areas include urban populations not located within an MSA as well as completely rural areas.

In analyses stratified by Census region, urbanicity, age, annual household income, and health insurance, similar racial and ethnic patterns emerged in most sociodemographic strata (Supplementary Figure, https://stacks.cdc.gov/view/cdc/118051). Asian adults had the highest coverage in all months since April 2021, and in November 2021, had the highest coverage across almost all sociodemographic categories, ranging from 87.2% among the uninsured to 98.1% in persons aged 50–64 years (Supplementary Table, https://stacks.cdc.gov/view/cdc/118052). By November 2021, coverage among Hispanic adults reached or exceeded that of White adults in all sociodemographic categories except those in the Midwest Census region (difference = −3.7). Differences in coverage among Black and NH/OPI adults were no longer present in November 2021, except in the Midwest (−5.4), urban areas (−6.6), and among those aged 18–29 years (−4.2) for Black adults, and in the Midwest (−24.3), South (−19.0), and among persons aged 18–29 years (−14.1) for NH/OPI adults.

Within racial and ethnic groups, coverage varied by subgroup. For example, among Asian adults, coverage ranged from 97.8% among persons identifying as Asian Indian, to 86.5% among other Asian persons (Table 2). Among Hispanic adults, coverage ranged from 90.6% among persons identifying as South American, to 79.3% among those identifying as Mexican. Coverage among Black adults was similar across subgroups (range = 73.6%–79.8%), with the exception of adults identifying as Somali (coverage = 52.6%).

**TABLE 2:** COVID–19 vaccination (≥1 dose) coverage estimates* among adults aged ≥18 years, by Asian, Hispanic, and Black subgroups^†^ — National Immunization Survey Adult COVID Module, United States, November 2021

Race and ethnicity	≥1-dose COVID-19 vaccination coverage, % (95% CI)
**Asian**	
Asian Indian	97.8 (96.6–98.7)
Chinese	95.2 (93.2–96.8)
Korean	94.2 (90.8–96.6)
Japanese	92.9 (88.0–96.4)
Filipino	92.4 (88.7–95.3)
Vietnamese	90.0 (84.9–94.0)
Other	86.5 (82.6–90.0)
**Hispanic**	
South American	90.6 (87.5–93.3)
Cuban	83.8 (77.2–89.3)
Puerto Rican	82.9 (80.6–85.1)
Central American	82.0 (78.3–85.3)
Mexican	79.3 (77.8–80.7)
Other	82.6 (79.4–85.6)
**Black**	
Jamaican	79.8 (73.4–85.5)
Nigerian	79.4 (72.9–85.3)
African American	77.7 (76.5–78.9)
Haitian	74.1 (62.5–84.3)
Ethiopian	73.6 (62.9–83.2)
Somali	52.6 (33.0–75.1)
Other	77.5 (73.8–81.0)

* Kaplan-Meier survival analysis was used to estimate vaccination coverage based on the month and year of first dose receipt; estimates reflect the cumulative percentage vaccinated as of the end of each month.

^†^ Persons were categorized into mutually exclusive categories of race and ethnicity; persons who did not identify as Hispanic were categorized by their reported race or races. For persons reporting they were Asian, Black, or Hispanic, an additional question was asked to determine a more specific ethnic or racial group.

## Discussion

During December 2020–November 2021, disparities in COVID-19 vaccination coverage among minority racial and ethnic groups narrowed. Disparities in COVID-19 age-adjusted mortality rates decreased during 2020–2021 for most racial and ethnic groups in the United States (*6*), likely related to reduced disparities in vaccination-related protection from COVID-19 infection. Substantial programmatic efforts to provide equitable access to COVID-19 vaccines might have contributed to closing the coverage gap. COVID-19 vaccines were made available free of charge at various providers and locations, including pharmacies, mass vaccination clinics, hospitals, and federally qualified health centers. CDC awarded supplemental funding to U.S. jurisdictions and other national, state, local, and community-level partner organizations to support efforts to increase coverage equity and access to vaccines, particularly among populations disproportionately affected by COVID-19, including racial and ethnic minority adults.^†††^

Differences in coverage by race and ethnicity within high and low socioeconomic strata suggest additional factors beyond access that led to disparities in vaccination. Although Hispanic adults were slower to be vaccinated, by the end of November 2021, this group had significantly higher coverage than did White adults across almost all categories assessed. Among adults who were uninsured or below the poverty level, COVID-19 vaccination coverage among Hispanic adults was >15 percentage points higher than that among White adults, suggesting that access issues typically associated with lower socioeconomic status were not necessarily barriers to vaccination among all racial and ethnic groups. Reported difficulty obtaining vaccine did not differ between Hispanic and White adults who were uninsured or below the poverty level; however, White adults in these groups reported more vaccine hesitancy, with approximately three times as many persons saying they definitely or probably would not get vaccinated (*7*).

As of October 31–December 31, 2021, 6.2% of White adults still intended to get vaccinated; among racial and ethnic minority groups, intent to get vaccinated was higher among NH/OPI (12.8%), Black (11.2%), AI/AN (10.3%), and Hispanic adults (9.3%)^§§§^ (*7*), indicating the potential for coverage to continue to increase among these groups. Analysis of the behavioral and social drivers of COVID-19 vaccination among those who were still unvaccinated, but willing to get vaccinated, during October 31–December 31, 2021, indicates that large proportions of AI/AN, Black, and multiple and other race adults are concerned about getting COVID-19 and think the vaccine is important and safe, yet they remain unvaccinated. For example, in the groups with continuing disparities (those in the Midwest and urban areas, and adults aged 18–29 years), fewer Black and Hispanic than White adults reported that they definitely or probably will not get vaccinated, indicating potential for increases in coverage among these groups with the appropriate interventions.

Whereas coverage among Asian adults exceeded 75% by May 2021, coverage among Hispanic and White adults did not reach this level until 4 months later (September), and until 5 months later (October) among Black and NH/OPI adults; coverage among AI/AN and multiple or other race adults remained <75% at the end of November 2021. Slower rates of vaccination by racial and ethnic minority groups likely resulted in potentially avoidable COVID-19 mortality in the interim, particularly among populations at higher risk for severe COVID-19–related outcomes or those who had increased occupational exposure risk because they were essential or frontline workers (*8*).

The findings in this report are subject to at least five limitations. First, response rates for NIS-ACM were relatively low (<25%), although similar to those in other NIS surveys.^¶¶¶^ Data were weighted to mitigate possible bias resulting from incomplete sample frame (i.e., exclusion of households with no phone service or only landline telephones) or nonresponse, but some selection bias might persist. Second, all responses were self-reported; vaccination receipt, and month and year of receipt of first dose might be subject to recall or social desirability bias. Third, the survey sampled noninstitutionalized U.S. adults; therefore, adults who were incarcerated or nursing home residents might not be represented in the sample. Fourth, although survey weights were calibrated to state-level vaccine administration data reported on CDC, NIS-ACM estimates of vaccination coverage might differ from vaccine administration data reported to CDC’s COVID Data Tracker.**** Finally, race and ethnicity information was missing for 2.9% of NIS-ACM respondents, compared with approximately 25% of vaccine administration records^††††^; coverage estimates for certain racial and ethnic groups might differ between the two sources because of differential omission of race and ethnicity information.

Equitable access to and receipt of COVID-19 vaccination is critical to reducing persistent disparities in vaccination coverage, morbidity, and mortality by race and ethnicity (*9*). Booster doses of COVID-19 vaccine are now recommended for all adults to boost immunity and improve protection against COVID-19 (*4*). Disparities in booster dose coverage among the fully vaccinated are becoming apparent (*5*), and the strategies that were successful in reducing disparities in primary dose COVID-19 vaccination could be applied to ensure equitable booster dose coverage. Tailored efforts including community partnerships and trusted sources of information could be used to increase vaccination coverage among the groups with identified persistent disparities and can help achieve vaccination equity and prevent new disparities by race and ethnicity in booster dose coverage.

**Summary**

**What is already known about this topic?**

Racial and ethnic minority groups have been disproportionately affected by the COVID-19 pandemic. Vaccination is effective in preventing COVID-19 infection and severe illness, and equitable vaccine administration can reduce COVID-19–related disparities.

**What is added by this report?**

Asian and non-Hispanic White adults had the highest COVID-19 vaccination coverage by the end of April 2021. By the end of November 2021, disparities in vaccination coverage for some racial and ethnic groups narrowed, and coverage was similar for non-Hispanic Black (78.2%), Hispanic (81.3%), Native Hawaiian and other Pacific Islander (75.7%), and non-Hispanic White (78.7%) adults.

**What are the implications for public health practice?**

Equitable access to and receipt of COVID-19 vaccination, including booster doses, is critical to reducing racial and ethnic disparities in vaccination.

## Footnotes

* Local areas that received federal immunization funds under Section 317 of the Public Health Service Act are sampled separately in NIS. Local areas include Bexar County, Texas; Chicago, Illinois; Houston, Texas; New York, New York; and Philadelphia County, Pennsylvania. Three U.S. territories were sampled separately in 2021: Guam (April–July 2021), Puerto Rico, and U.S. Virgin Islands.

† Survey weights were calibrated to the COVID-19 vaccine administration data by jurisdiction, age group, and sex. https://covid.cdc.gov/covid-data-tracker/ (Accessed May 26, 2022).

§ Race and ethnicity were assessed by the following two questions: “Are you of Hispanic or Latino origin?” and “Now, I am going to read a list of categories. Please choose one or more of the following categories to describe your race. Are you White, Black or African American, American Indian, Alaska Native, Asian, Native Hawaiian or other Pacific Islander?” Persons were categorized into mutually exclusive categories of race and ethnicity; persons who did not identify as Hispanic were categorized by their reported race or races. For persons reporting they were Asian, Black, or Hispanic, an additional question was asked to determine a more specific ethnic or racial group.

¶ Receipt of ≥1 dose of COVID-19 vaccine was assessed by the question, “Have you received at least one dose of a COVID-19 vaccine?” Month and year of first dose was assessed by the question, “During what month and year did you receive your first COVID-19 vaccine?” Respondents who were interviewed during April 22–December 31, 2021, were included in this study; month and year of vaccination could be before the study period.

** Calculated according to the American Association for Public Opinion Research type 3 response rate. https://www.aapor.org/AAPOR_Main/media/publications/Standard-Definitions20169theditionfinal.pdf

†† Kaplan-Meier methods were used to calculate cumulative monthly vaccination estimates during December 2020–November 2021. Vaccination status was assigned using the reported month and year of the first dose of COVID-19 vaccine, as of the end of the month before interview.

§§ *Northeast*: Connecticut, Maine, Massachusetts, New Hampshire, New Jersey, New York, Pennsylvania, Rhode Island, and Vermont; *Midwest*: Illinois, Indiana, Iowa, Kansas, Michigan, Minnesota, Missouri, Nebraska, North Dakota, Ohio, South Dakota, and Wisconsin; *South*: Alabama, Arkansas, Delaware, District of Columbia, Florida, Georgia, Kentucky, Louisiana, Maryland, Mississippi, North Carolina, Oklahoma, South Carolina, Tennessee, Texas, Virginia, and West Virginia; *West*: Alaska, Arizona, California, Colorado, Hawaii, Idaho, Montana, Nevada, New Mexico, Oregon, Utah, Washington, and Wyoming.

¶¶ Urbanicity was determined based on household reported city and county of residence and was grouped into three categories: metropolitan statistical area (MSA) principal city (urban), MSA nonprincipal city (suburban), and non-MSA (rural). MSAs and principal cities were categorized as defined by the U.S. Census Bureau. https://www.census.gov/programs-surveys/metro-micro.html

*** 45 C.F.R. part 46, 21 C.F.R. part 56; 42 U.S.C. Sect. 241(d); 5 U.S.C. Sect. 552a; 44 U.S.C. Sect. 3501 et seq.

†††
https://www.cdc.gov/vaccines/health-equity/index.html

§§§ Those who said they would “definitely get a vaccine,” “probably get a vaccine,” or were “not sure” were considered to have intent to get vaccinated.

¶¶¶
https://www.cdc.gov/vaccines/imz-managers/nis/data-tables.html

**** https://covid.cdc.gov/covid-data-tracker/#vaccinations_vacc-total-admin-rate-total (Accessed May 26, 2022).

†††† https://covid.cdc.gov/covid-data-tracker/#vaccination-demographic (Accessed May 26, 2022).

## References

[1] Mackey K, Ayers CK, Kondo KK, Racial and ethnic disparities in COVID-19–related infections, hospitalizations, and deaths: a systematic review. Ann Intern Med 2021;174:362–73. 10.7326/M20-6306 33253040 PMC7772883

[2] Bilal U, Jemmott JB, Schnake-Mahl A, Murphy K, Momplaisir F. Racial/ethnic and neighbourhood social vulnerability disparities in COVID-19 testing positivity, hospitalization, and in-hospital mortality in a large hospital system in Pennsylvania: a prospective study of electronic health records. Lancet Reg Health Am 2022;10:100220. 10.1016/j.lana.2022.100220 35262038 PMC8891851

[3] Tenforde MW, Self WH, Gaglani M, ; IVY Network. Effectiveness of mRNA vaccination in preventing COVID-19–associated invasive mechanical ventilation and death—United States, March 2021–January 2022. MMWR Morb Mortal Wkly Rep 2022;71:459–65. 10.15585/mmwr.mm7112e1 35324878 PMC8956334

[4] Mbaeyi S, Oliver SE, Collins JP, The Advisory Committee on Immunization Practices’ interim recommendations for additional primary and booster doses of COVID-19 vaccines—United States, 2021. MMWR Morb Mortal Wkly Rep 2021;70:1545–52. 10.15585/mmwr.mm7044e2 34735422 PMC8568093

[5] Fast HE, Zell E, Murthy BP, Booster and additional primary dose COVID-19 vaccinations among adults aged ≥65 years—United States, August 13, 2021–November 19, 2021. MMWR Morb Mortal Wkly Rep 2021;70:1735–9. 10.15585/mmwr.mm7050e2 34914672 PMC8675661

[6] Truman BI, Chang MH, Moonesinghe R. Provisional COVID-19 age-adjusted death rates, by race and ethnicity—United States, 2020–2021. MMWR Morb Mortal Wkly Rep 2022;71:601–5. 10.15585/mmwr.mm7117e2 35482556 PMC9098236

[7] Kriss JL, Hung MC, Srivastav A, Intent to receive COVID-19 vaccine and behavioral and social drivers of vaccination by race and ethnicity, National Immunization Survey Adult COVID Module—United States, October 31–December 31, 2021. Atlanta, GA: US Department of Health and Human Services, CDC; 2022. https://www.cdc.gov/vaccines/imz-managers/coverage/covidvaxview/pubs-resources/intent-receive-covid19-vaccine-behavioral-social-drivers.html

[8] McClure ES, Vasudevan P, Bailey Z, Patel S, Robinson WR. Racial capitalism within public health—how occupational settings drive COVID-19 disparities. Am J Epidemiol 2020;189:1244–53. 10.1093/aje/kwaa126 32619007 PMC7337680

[9] Wong CA, Dowler S, Moore AF, COVID-19 vaccine administration, by race and ethnicity—North Carolina, December 14, 2020–April 6, 2021. MMWR Morb Mortal Wkly Rep 2021;70:991–6. 10.15585/mmwr.mm7028a2 34264909 PMC8314707

